# Special Issue “Microbial Interactions in Soil”: Editorial

**DOI:** 10.3390/microorganisms11051260

**Published:** 2023-05-11

**Authors:** Volker S. Brozel

**Affiliations:** 1 Department of Biology and Microbiology, South Dakota State University, Brookings, SD 57006, USA; volker.brozel@sdstate.edu; 2 Department of Biochemistry, Genetics and Microbiology, University of Pretoria, Pretoria 0004, South Africa

Soils are home to a wide variety of microorganisms. The collective genome of these organisms is vast, and so individual strains perform some functions that few others can. In addition to the consumption of specific metabolites, many soil microbes excrete unique compounds into their direct environment in soils, at root surfaces, and inside plants as endophytes. The ensuing localized build-up of compounds impacts other organisms by exerting a variety of functions: promoting growth, activating signal transduction, suppressing cellular functions, or changing the physicochemical environment. Microorganisms share soils with plants, whose roots exude cocktails of organic compounds [1,2] that further impact the surrounding microbiota and their ensuing interactions. This Special Issue shares findings on three higher levels of interactions, and how these can affect microbial diversity:

- Plant genotype influences interactions among associated bacteria and fungi;
- Plant-growth-promoting bacterial communities lead to outcomes exceeding the sum of individual effects; and
- Environmental conditions impact how microbes in communities interact.

Plant breeding has focused largely on improved agronomic traits, such as yield and disease resistance, invariably losing some parental traits not selected for. Rhizomicrobiome analysis of modern cultivars versus ancestral accessions has revealed significant differences in the bacterial and fungal communities of rice [3]. The progeny of crosses between female parent *Oryza rufipogon* wild rice and male parent *Oryza sativa* cultivated rice had significantly different bacterial and fungal communities than either of the parental lines. Similarly, sorghum lines have different rhizo- and endosphere bacterial communities that contribute to plant performance to different degrees under low nitrogen stress conditions [4]. Cultivation of sugarcane leads to shifts in both fungal and bacterial community composition in soil. These microbial community shifts were associated with decrease in yield [5].

A unique three-way interaction has been reported among plants, their endophytes, and the associated rhizomicrobial community. Fungal endophytes of Tall Fescue are not only associated with improved biomass yield and stress tolerance, but also with soil fungal community composition and increased diversity [6]. These shifts were associated with enhanced phosphate availability.

Physicochemical conditions are well known to affect the growth of individual microbial cultures. Invariably, shifts in environmental conditions also impact the fitness of individual species and strains. Farda et al. reviewed the literature on actinomycetes in caves [7]. The scarcity of carbon in cave environments acts as a unique evolutionary stressor, leading to unique biosynthetic capabilities in these actinomycetes. Similarly, heavy-metal-containing soils were reported to contain novel actinobacteria, including *Streptomyces* [8]. Tarin et al. outlined shifts in soil fungal microbiota after the incorporation of bamboo biochar into soil [9].

Various microbial strains isolated from soils have been shown to benefit crop production through mechanisms such as nutrient acquisition or suppression of pathogens. Wang et al. present an analysis of how the consortia of disease-suppressing microorganisms bring about enhanced protection when compared to individual strains [10]. They go on to point out criteria for selecting specific disease-suppressive strains to identify effective consortia.

The contributions to this Special Issue all point to the complexity of specific interactions that lead to shifts in microbial communities under specific conditions. Much remains to be done to unravel the specific components of these interactions.
